# Supplementary figures and images for: Paeniclostridium sordellii uterine infection is dependent on the estrous cycle
Source: PLoS Pathog. 2022 Nov 21;18(11):e1010997. doi: 10.1371/journal.ppat.1010997 (PMC9721474; doi:10.1371/journal.ppat.1010997)

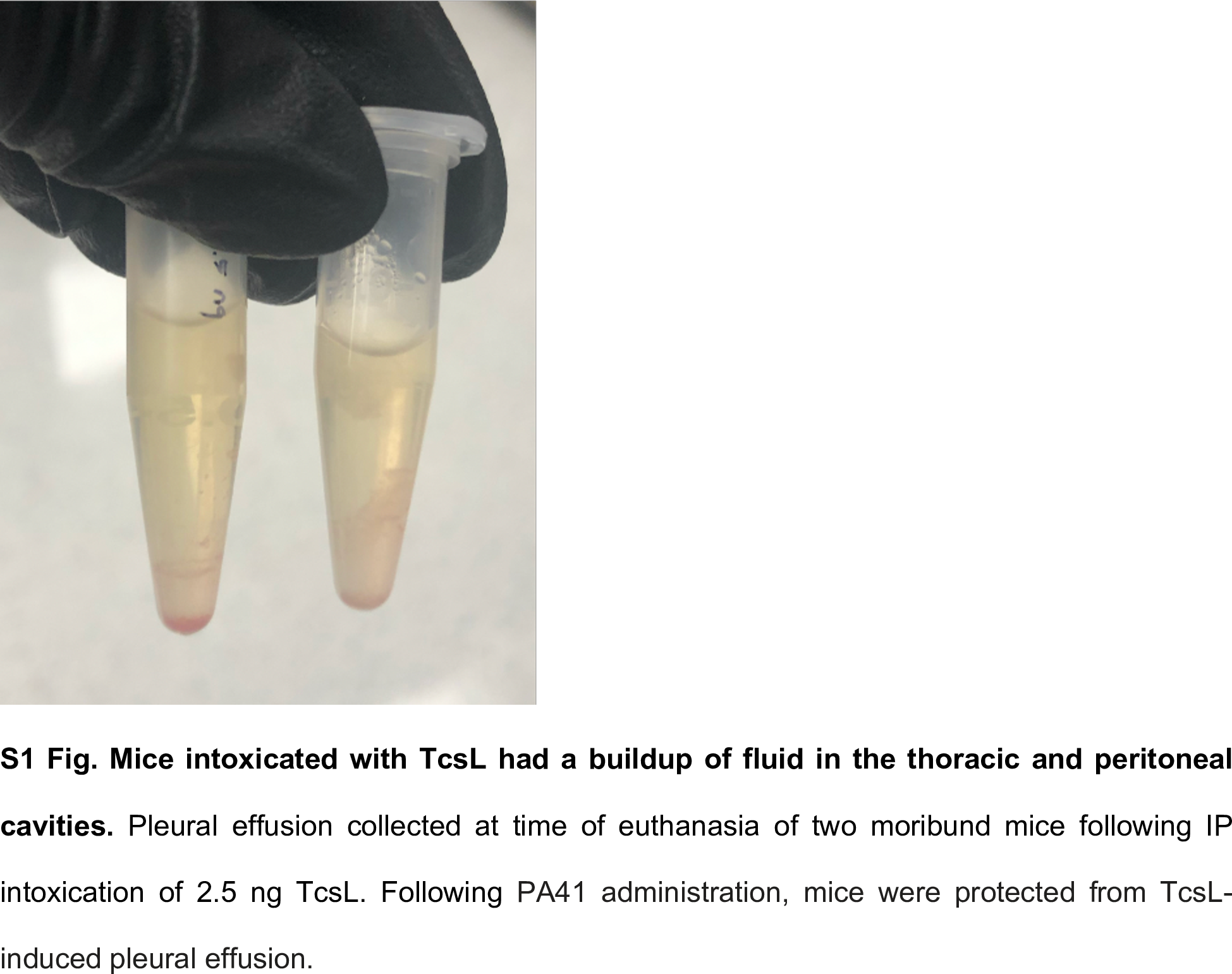

Supplement: S1 Fig — Pleural effusion collected at time of euthanasia of two moribund mice following IP intoxication of 2.5 ng TcsL. Following PA41 administration, mice were protected from TcsL-induced pleural effusion. (TIF) [file ppat.1010997.s001.tif]

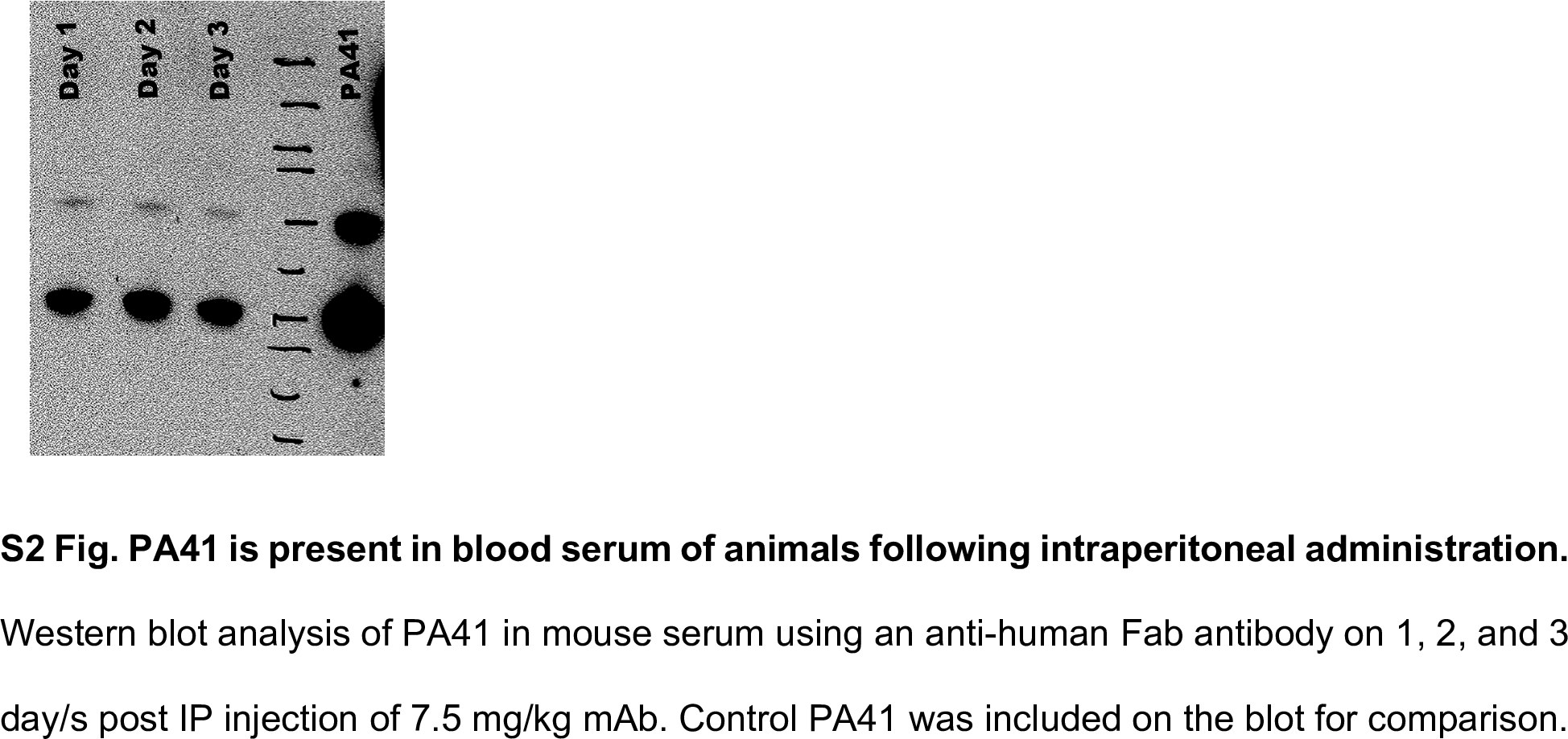

Supplement: S2 Fig — Western blot analysis of PA41 in mouse serum using an anti-human Fab antibody on 1, 2, and 3 days post IP injection of 7.5 mg/kg mAb. Control PA41 was included on the blot for comparison. (TIF) [file ppat.1010997.s002.tif]

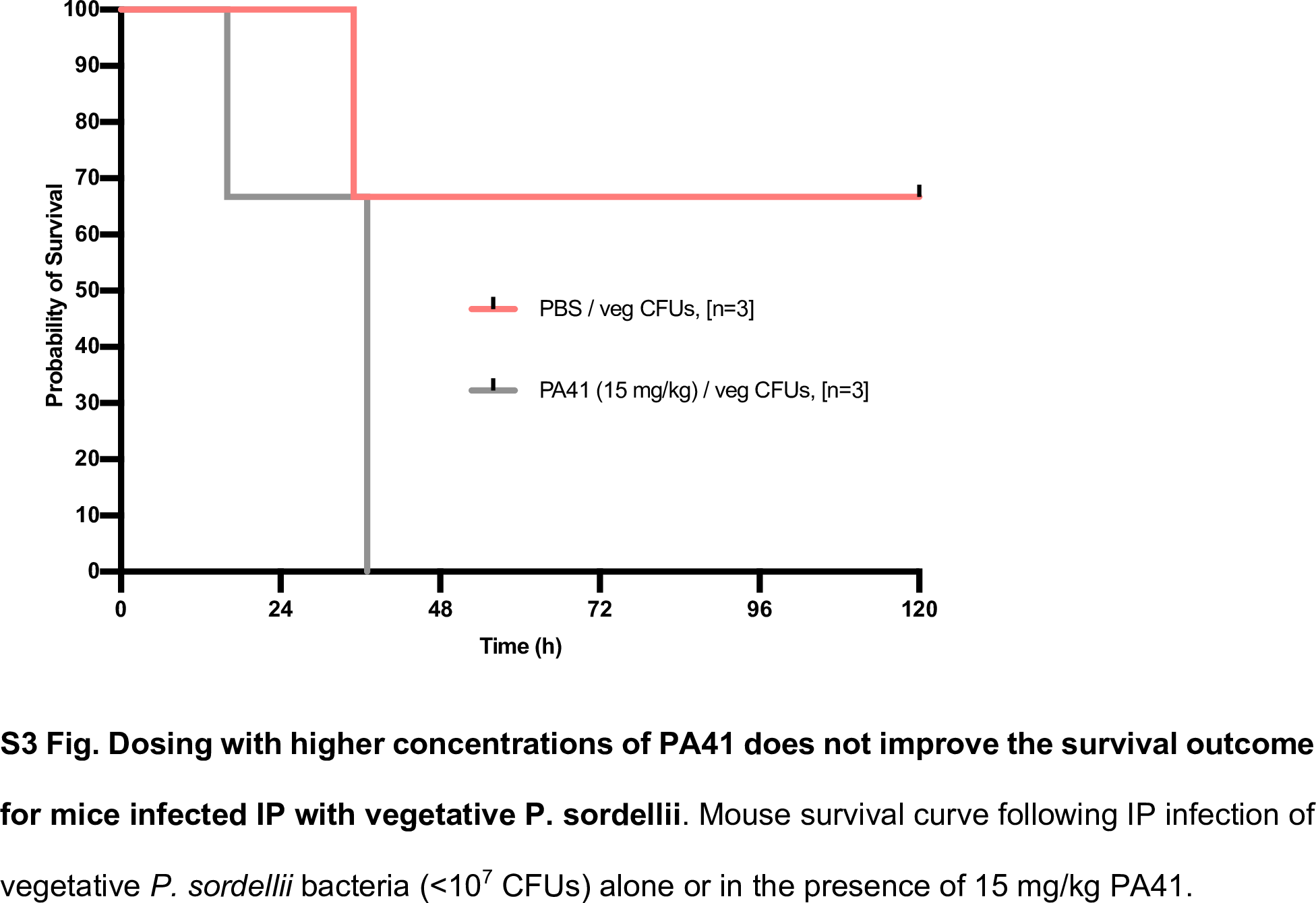

Supplement: S3 Fig — Mouse survival curve following IP infection of vegetative P. sordellii bacteria (<107 CFUs) alone or in the presence of 15 mg/kg PA41. (TIF) [file ppat.1010997.s003.tif]

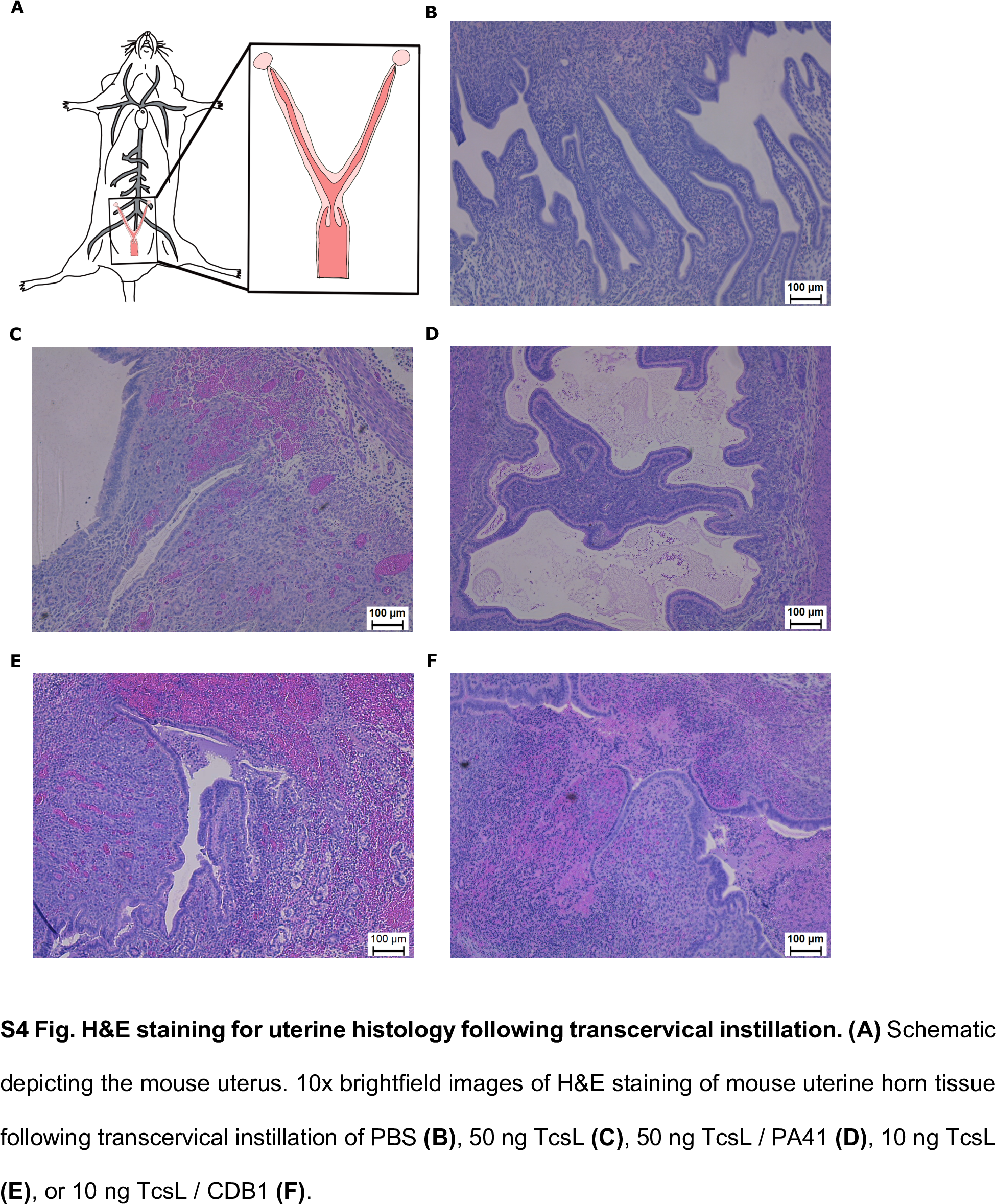

Supplement: S4 Fig — (A) Schematic depicting the mouse uterus. 10x brightfield images of H&E staining of mouse uterine horn tissue following transcervical instillation of PBS (B), 50 ng TcsL (C), 50 ng TcsL / PA41 (D), 10 ng TcsL (E), or 10 ng TcsL / CDB1 (F). (TIF) [file ppat.1010997.s004.tif]

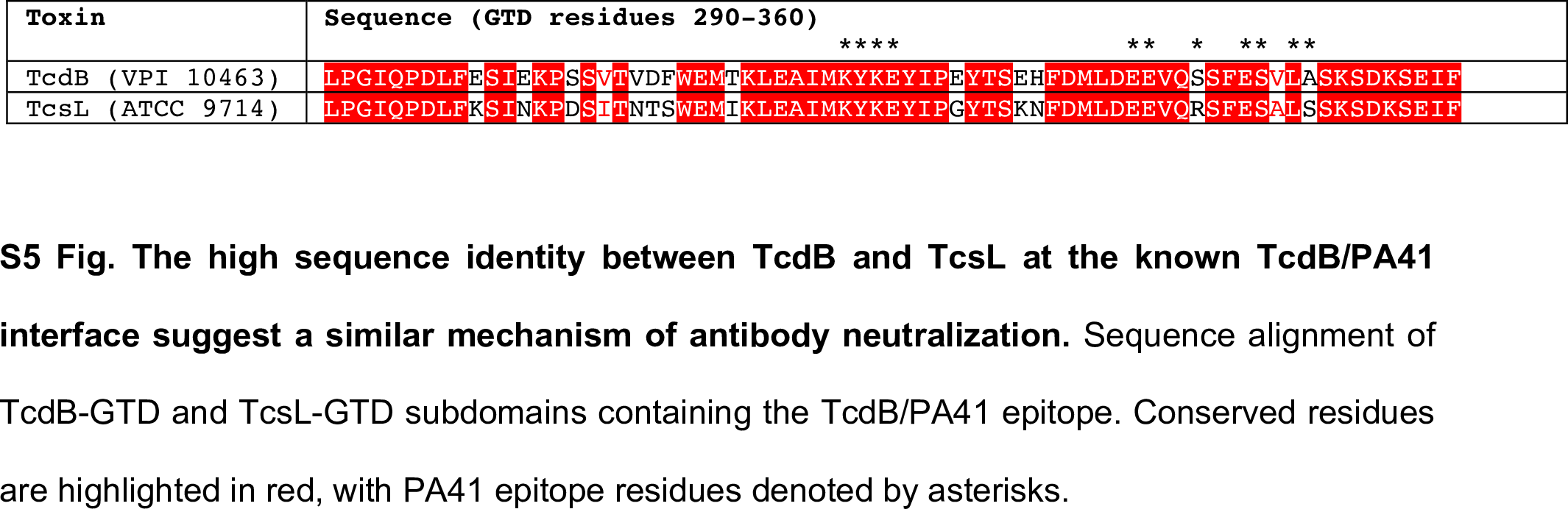

Supplement: S5 Fig — Sequence alignment of TcdB-GTD and TcsL-GTD subdomains containing the TcdB/PA41 epitope. Conserved residues are highlighted in red, with PA41 epitope residues denoted by asterisks. (TIF) [file ppat.1010997.s005.tif]
