# Supplementary material for: Paeniclostridium sordellii uterine infection is dependent on the estrous cycle
Source: PLoS Pathog. 2022 Nov 21;18(11):e1010997. doi: 10.1371/journal.ppat.1010997 (PMC9721474; doi:10.1371/journal.ppat.1010997)
Supplement: Attachment — Submitted filename: PSI_Response to comments_093022.pdf [file ppat.1010997.s006.pdf]

September 30, 2022

To the editor:

Thank you for coordinating the review of our manuscript, PPATHOGENS-D-22-01162. We are happy to have the positive and constructive feedback and have prepared a revised manuscript taking that feedback into consideration. Here we provide a point-by-point response to the reviewer comments and a description of the changes made in the manuscript.

<u>Major Issue Comments from Reviewer 1</u>

- **Comment**: *Considering how straightforward the in vitro experiments are to perform, as compared with the in vivo experiments, it is surprising that the authors did not provide a more complete set of experiments in Figure 1. In particular, the authors should include a dose titration of both antibodies (at a cytotoxic dose of TcsL) to get a sense of their relative potencies.*

  Response: We agree with this point and have modified Figure 1 to include a dose titration of both antibodies at a cytotoxic concentration of 1 pM TcsL (Fig. 1C-D).

- **Comment**: *Furthermore, it would be desirable if the data of the titration of toxin in the presence and absence of a fixed dose of antibody were presented more clearly. A full titration of toxin, perhaps with small increments shown as curves plus and minus antibody would be preferable. The inclusion of red bar for No AB is confusing also.*

  Response: In Figure 1A, we now show a full titration of toxin as a curve with and without the presence of PA41 or CDB1. We have chosen to also include the same data as a bar graph in Figure1B, as this clearly shows the TcsL concentrations where we see statistically significant differences. We have also retained a red color for the treatments without antibody to aid with visualizing differences.

- **Comment**: *[optional] In Figure 5, rather than pre-dose animals with antibodies before the toxin challenge, did the authors consider dosing the antibodies right before or right after the TC challenges were performed. Without any knowledge of the PK of the antibodies, it is difficult to know how much antibody is circulating. The efficacy might be better if dosed differently.*

  Response: We did consider dosing the antibody at different times and, in response to other comments, we try to now provide the reader with a narrative explaining the decisions we made on dosing over the course of these studies (lines 127-139 and lines 206-211). For the intoxication experiment, we used sequential dosing of antibody prior to intoxication to give the animals the best chance at survival. TcsL is rapidly lethal, and we were concerned that there would not be enough time to use our mAbs for treatment following intoxication. We saved the mAB treatment approaches for the more physiological infection experiments. (Figure 6 D and E).
  With regard to the question of the antibody pharmacokinetics, we are now showing in S2 Fig that following a single IP injection of PA41 at 7.5 mg/kg, the antibody remains within the bloodstream for three days without any signs of depletion. We therefore do not believe that antibody efficacy is being limited by pharmacokinetic depletion in the time course of our experiments.

- **Comment**: *The marginal efficacy seen in Figure 6E in "treatment mode" was underwhelming. Did the authors consider dosing higher? There was no specific rationale given for dosing at 7.5mpk. If it is not dose-limiting, it would be important to explore whether a higher dose of antibody might work in this paradigm.*

  Response: As indicated above, we now provide the rationale for the decisions we made regarding the timing, frequency, and concentration of antibody used in the different intoxication and infection experiments (lines 127-139 and lines 206-211). We started the sequential dosing of 7.5 mg/kg mAB, because we found that a single IP of 15 mg/kg worsened infection. This is now described in lines 206-211, and shown in S3 Fig.

  While we do understand that the efficacy of PA41 can likely be improved with further optimization of the experimental variables, there will be limitations in that we are using humanized antibodies in a mouse model of infection. A deeper exploration of protection in the mouse would require converting these antibodies to mouse frameworks and is outside the scope of this study. Our objective has been to test for any indication of a protective effect in mice that could then be used to justify studies in humans. We feel that these studies provide the proof of concept that cross-neutralizing antibodies in development for use in *C. difficile* infection could have expanded utilities in the rare cases of *P. sordellii* infection.

<u>Major Issue Comments from Reviewer 2</u>

- **Comment**: *There are many antibodies/nanobodies/immune molecules developed that can effectively neutralize TcdB. But only PA41 and CDB1 were tested. The authors should give a more comprehensive evaluation of other known TcdB antibodies/nanobodies/immune molecules and compare their neutralizing effects against TcsL.*

  Response: Thank you for this suggestion. It would have been interesting to explore additional TcdB neutralizers against TcsL. However, in the case of our study, we did not intend it to be a comprehensive evaluation of TcsL neutralization, but to showcase the use of our hormone-inducing uterine model for evaluating therapeutics for *P. sordellii* infections. For this study, we chose CDB1 and PA41 for their potential clinical availability. CDB1 is already FDA approved and available for clinical use under the tradename Zinplava, and PA41 is being developed for the treatment of CDI by Astra Zeneca.

- **Comment**: *The neutralizing effects of PA41 and CDB1 are not characterized quantitively. What are the IC50 values in the cell models. Also, multiple cell lines need to be tested. What are the binding affinities (KD) of these antibodies to TcsL, and compared to TcdB?*

  Response: We now provide IC_{50} values for the antibodies in Figure 1C-D. PA41 and CDB1 neutralize the cytotoxic effects of 1 pM TcsL on Vero cells with IC_{50}s of 20 pM and ~41 nM, respectively. As indicated above, our focus in this study was to develop a physiological model for the study of PSI *in vivo*. While we agree that studies of potency on different cell types can be valuable, especially when exploring foundational questions of receptor binding, the question in this study was more practical: does a clinically available (CDB1) or potentially available (PA41) therapeutic provide protection in this newly established model of transcervical infection. We therefore elected to focus this study on the *in vivo* efficacy of these antibodies.

Comment: *P. sordellii produces two major exotoxins, TcsL and TcsH. Although less toxic, TcsH causes strong hemorrhagic effects and should not be ignored. A combination of neutralizing agents against both TcsL and TcsH may bring optimized protection against P. sordellii. In particular, a recent study has defined TMPRSS2 as a receptor for TcsH, which can help to design neutralizing molecules against TcsH.*

Response: We agree that the role of TcsH when present is quite interesting and should not be ignored. However, many virulent *P. sordellii* strains lack TcsH, and multiple papers, including this one, provide evidence that it is not required for causing disease. For this study, we are using a highly virulent reference strain (ATCC 9714) of *P. sordellii* that lacks TcsH. We have made a modification to the introduction to make that point clearer (lines 73-75).

- **Comment**: *In the previous studies, lung was proposed as a vulnerable target for TcsL. Why was this lung damage model was not used to evaluate the protection effect of PA41? Also, studies showed that soluble SEMA6A/B can effectively protected mouse from TcsL-induced lung damage. What is the neutralizing efficacy of PA41 compared to SEMA6A/B in vivo?*

  Response: The lung damage model is an intraperitoneal injection of TcsL, and the data presented in Figure 2 uses this model. All mice intoxicated with TcsL had a buildup of fluid in the lungs. In contrast, antibody administration protected mice from TcsL-induced pleural effusion. We have added this result into the manuscript (lines 123-125, 139-140) and have included in S1 Fig an image of the thoracic fluid. We chose not to optimize this animal model further because we believe our transcervical model is more physiologically relevant for women experiencing uterine *P. sordellii* infection.

  With regard to the use of SEMA6A/B, we agree that this could be another strategy for protecting against the lethal effects of TcsL. However, as indicated above, we did not intend for this to be a comprehensive evaluation of TcsL neutralization, but to showcase the use of our hormone-inducing uterine model for evaluating therapeutics for *P. sordellii* infections.

- **Comment**: *In transcervical infection models, the difference of TcsL/P. sordellii susceptibility between mice in diestrus and estrus may simply be explained by morphological and physiological changes of cervix. For example, minor wounds and increased permeability of the epithelium during estrus. To demonstrate this (or not), more pathological and biochemical analysis on cervix need to be performed.*

  Response: We do not believe the cervix is playing a strong role in our model as we bypass the cervix and instill directly into one of the uterine horns. Histopathologically, we do not observe any cervical damage or changes following transcervical intoxication or infection. We agree though that the difference of TcsL and *P. sordellii* susceptibility between mice in estrus and diestrus is likely due to morphological and physiological changes in the uterine tissue that occur at these two different stages of the cycle. We speculate that the epithelial remodeling and increased permeability of the epithelium associated with diestrus, and not estrus, is allowing TcsL access to the bloodstream. In addition, it is known that in estrus there is an increased production of mucus in the uterus. Presumably, this could give the animals a layer of protection preventing toxin from reaching the epithelium of the uterus. We have added these ideas to our discussion (lines 296-301).

- **Comment**: *Also, IP injection of TcsL/P. sordellii in mice at diestrus and estrus state should be performed as a comparison.*

  Response: IP injections of TcsL and *P. sordellii* result in all animals dying regardless of the reproductive cycles as shown in Fig 2A and C.

Minor Comments from Reviewer 1

- **Comment**: *The title of the paper is too vague. It sounds like a review paper title. Please fix this to make it more informative.*

  Response: We have changed the title of the paper to reflect the major finding of this study.

- **Comment**: *The authors should provide some rationale for the doses of antibodies used throughout this study (0.75mpk ->7.5mpk)*

  Response: We have included rationale for the doses of antibodies. See lines 127-139 and lines 206-211.

- **Comment**: *The data in Figure 3D are marginal. The language on Line 141-142 could be softened.*

  Response: We agree and have made these adjustments. See now lines 157-159.

Minor Comments from Reviewer 2

- **Comment**: *The mechanism of virulence is merely mentioned, and the title is overstated*.

  Response: We have changed the title of the paper to reflect the findings more accurately.

- **Comment** *The numbers of mice used in some groups were not sufficient to obtain reliable conclusions, such as TcsL/CDB1 in Fig2B and IP-106 in Fig2C.*

  Response: We agree with this point and have been careful not to over interpret these data. We include the data from IP pilot studies as they were important in the path we took to define experimental conditions, but our primary goal was to get to the uterine infection model. To minimize the number of experimental animals, we took a conservative approach if we knew we would want to repeat the experiment with vegetative bacteria and/or spores. Key experiments were performed at least twice with group sizes that would permit statistical analyses and reliable conclusions.

- **Comment**. *Since co-structure of PA41-TcdB is known (actually from the same group), it would be ideal to compare the consensual and divergent residues at the interface between PA41-TcdB and PA41-TcsL.*

  Response: We have now included in S5 Fig an alignment of TcdB and TcsL at the PA41 epitope and point out in lines 270-272 that the epitope is largely conserved.

- **Comment**. *Fig 3. How did the author control the estrous cycle stage of the mice here?*

Response: For the experiments that are represented in Fig 3, we did not regulate the estrous cycle and the animals were cycling naturally. We speculate that the animals that succumbed to disease were in a stage of the reproductive cycle that is more susceptible (i.e., diestrus) while the resistant animals were likely in a stage of the cycle that conveys protection (i.e., estrus).
